# In Alzheimer’s Disease, 6-Month Treatment with GLP-1 Analog Prevents Decline of Brain Glucose Metabolism: Randomized, Placebo-Controlled, Double-Blind Clinical Trial

**DOI:** 10.3389/fnagi.2016.00108

**Published:** 2016-05-24

**Authors:** Michael Gejl, Albert Gjedde, Lærke Egefjord, Arne Møller, Søren B. Hansen, Kim Vang, Anders Rodell, Hans Brændgaard, Hanne Gottrup, Anna Schacht, Niels Møller, Birgitte Brock, Jørgen Rungby

**Affiliations:** ^1^Institute of Biomedicine, Aarhus UniversityAarhus, Denmark; ^2^Department of Nuclear Medicine and PET Center, Aarhus University HospitalAarhus, Denmark; ^3^Department of Neuroscience and Pharmacology, University of CopenhagenCopenhagen, Denmark; ^4^Dementia Clinic, Department of Neurology, Aarhus University HospitalAarhus, Denmark; ^5^Department of Endocrinology, Aarhus University HospitalAarhus, Denmark; ^6^Department of Clinical Biochemistry, Aarhus University HospitalAarhus, Denmark; ^7^Center for Diabetes Research and Department of Clinical Pharmacology, Copenhagen University Hospital Gentofte and RigshospitaletCopenhagen, Denmark

**Keywords:** Alzheimer’s disease, amyloid, cerebral glucose metabolism, glucagon-like peptide-1, liraglutide

## Abstract

In animal models, the incretin hormone GLP-1 affects Alzheimer’s disease (AD). We hypothesized that treatment with GLP-1 or an analog of GLP-1 would prevent accumulation of Aβ and raise, or prevent decline of, glucose metabolism (CMR_glc_) in AD. In this 26-week trial, we randomized 38 patients with AD to treatment with the GLP-1 analog liraglutide (*n* = 18), or placebo (*n* = 20). We measured Aβ load in brain with tracer [^11^C]PIB (PIB), CMR_glc_ with [^18^F]FDG (FDG), and cognition with the WMS-IV scale (ClinicalTrials.gov NCT01469351). The PIB binding increased significantly in temporal lobe in placebo and treatment patients (both *P* = 0.04), and in occipital lobe in treatment patients (*P* = 0.04). Regional and global increases of PIB retention did not differ between the groups (*P* ≥ 0.38). In placebo treated patients CMR_glc_ declined in all regions, significantly so by the following means in precuneus (*P* = 0.009, 3.2 μmol/hg/min, 95% CI: 5.45; 0.92), and in parietal (*P* = 0.04, 2.1 μmol/hg/min, 95% CI: 4.21; 0.081), temporal (*P* = 0.046, 1.54 μmol/hg/min, 95% CI: 3.05; 0.030), and occipital (*P* = 0.009, 2.10 μmol/hg/min, 95% CI: 3.61; 0.59) lobes, and in cerebellum (*P* = 0.04, 1.54 μmol/hg/min, 95% CI: 3.01; 0.064). In contrast, the GLP-1 analog treatment caused a numerical but insignificant increase of CMR_glc_ after 6 months. Cognitive scores did not change. We conclude that the GLP-1 analog treatment prevented the decline of CMR_glc_ that signifies cognitive impairment, synaptic dysfunction, and disease evolution. We draw no firm conclusions from the Aβ load or cognition measures, for which the study was underpowered.

## Introduction

Type 2 diabetes (T2D) raises the risk of Alzheimer’s disease (AD) (Xu et al., 2009). Suggested common pathophysiological mechanisms include deficient insulin and glucagon-like peptide-1 (GLP-1) signaling (Femminella and Edison, 2014). GLP-1 stimulates β-cell neogenesis, growth, and differentiation, and inhibits β-cell apoptosis (Nauck, 2004). In the pancreas, β-cells secrete amylin that shares characteristics with Aβ. In animal models of T2D, β-cells accumulate amyloid, and GLP-1 treatment of the model animals relieves amyloid toxicity (Ahren et al., 2007). Cells of hypothalamus and hippocampus abundantly express GLP-1 receptors and GLP-1 induces neurite outgrowth (Perry and Greig, 2005). GLP-1 protects against excitotoxic cell death and against the toxic effects of Aβ_1-42_ by binding to receptors expressed in soma and dendrites of large neurons (Perry and Greig, 2005). In mice, intraventricular administration of GLP-1 reduces nerve cell damage triggered by neurotoxic stimuli, and GLP-1 receptor activation improves learning and memory (During et al., 2003). Treatment of mice with the GLP-1 analog liraglutide in an AD model halted the progression of decline in memory function (Hansen et al., 2015).

Current AD treatments remain symptomatic, however. GLP-1 receptor agonism potentially reverses both early and late events in neurodegeneration (McClean et al., 2011, 2015; McClean and Holscher, 2014). In a 1-year study of deep brain stimulation of patients with AD, a persistent increase of cerebral glucose metabolism was associated with improved clinical outcome and cortical circuitry compared to a 1-year course of pharmacotherapy (Smith et al., 2012). In humans, GLP-1 alters brain glucose transport and metabolism (Lerche et al., 2008; Gejl et al., 2012, 2013). Liraglutide is a GLP-1 receptor agonist used in T2D and chronic weight management. The analog crosses the blood–brain barrier into brain where it raises synaptic plasticity (McClean et al., 2010; Hunter and Holscher, 2012). GLP-1 mimetics are reported to be neuroprotective in a range of neurodegenerative disorders (Holscher, 2014). In the transgenic APP/PS1 mouse model of AD, liraglutide stimulates neuronal proliferation, improves learning, reduces plaque formation, and Aβ synthesis, inhibits inflammation, and raises neurogenesis (McClean et al., 2011).

In this first-in-human study, on the basis of the results of GLP-1 analog treatment and the effects on brain glucose metabolism, we examined the effects of liraglutide on three vital signs in AD, cerebral glucose consumption, fibrillary Aβ deposition, and cognition. We tested the hypothesis that treatment with liraglutide prevents or reduces the decline of cerebral glucose consumption measured with PET of [^18^F]fluoro-2-deoxyglucose ([^18^F]FDG) metabolism. We also tested whether 6 months’ treatment with liraglutide would reduce the Aβ deposition in brain of patients with AD, determined by PET of carbon-11 labeled Pittsburgh Compound B ([^11^C]PIB) retention. In parallel, we determined commensurate changes of relevant cognitive scores.

## Materials and Methods

### Study Design and Participants

We completed a 26-week, randomized, placebo-controlled, double-blinded intervention with liraglutide (VICTOZA^®^) or placebo in patients with AD. Patients were recruited from dementia clinics in Central Denmark, with key inclusion and exclusion criteria listed in **Table 1**. Patients willing to participate gave written informed consent. Safety data were monitored independently throughout the study period. The study was conducted according to the principles of the Helsinki Declaration. The Central Denmark Regional Committees on Biomedical Research Ethics, the Danish Data Protection Agency, and the Danish Medicines Agency approved the protocol, (Egefjord et al., 2012) with trial registration at ClinicalTrials.gov: NCT01469351.

**Table 1:** Key inclusion and exclusion criteria.

**Key inclusion criteria**
Adult competent persons.
Diagnosed with Alzheimer’s disease (AD) with an MMSE score of 18–21.
The diagnosis should be entirely based on clinical findings, while diagnosis by MMSE with a score >22 should be diagnosed by spinal puncture.
Age >50 years and <80 years.
Caucasians.
**Key exclusion criteria**
Diabetes mellitus.
Clinically significant liver or renal impairment (serum alanine amino transferase >2 times upper reference or creatinine-clearance <60 ml/min, assessed on Cockcroft-Gault normogram).
Clinically significant anemia.
Current or former presence of one of the following diseases with clinical relevance: another central nervous system illness other than diagnosed depression treated with SSRI or SSRI similar drugs; liver disease; kidney disease; endocrinological disease other than well- controlled hypothyroidism.
Current or history of chronic or acute pancreatitis.
Patients treated with tricyclic antidepressants or neuroleptics.
Significant abnormalities in the brain detected by magnetic resonance imaging.


*MMSE, Mini-Mental State Examination; SSRI, selective serotonin re-uptake inhibitors.*

### Randomization and Masking

Participants were randomly assigned (1:1) by block randomization using block size of eight by The Hospital Pharmacy, Central Denmark Region, Aarhus, Denmark. Study drugs were given in coded drug packages. Investigators and coordinators used a centralized code system for study package administration. Investigators dispensed study drug or matching placebo in pre-filled identically appearing pens. Participants, carers and study staff were masked to treatment assignment. Liraglutide or placebo was administered as “add on” to the patients’ usual medications, including already initiated and stabilized treatment with cholinesterase-inhibitors for AD.

### Procedures

Participants attended a screening visit to assess eligibility followed by randomization to either liraglutide or placebo for 26 weeks. Liraglutide was administered as 0.6 mg subcutaneously daily for 1 week; hereafter 1.2 mg daily for 1 week before finally increasing to 1.8 mg daily. Placebo was saline in similar volumes.

### Positron Emission Tomography

The subjects underwent PET with [^11^C]PIB and [^18^F]FDG. We synthesized [^11^C]PIB as previously described (Solbach et al., 2005). PET with [^11^C]PIB followed previous [^11^C]PIB studies of brain (Leinonen et al., 2008), apart from abbreviated recording time. Also PET with [^18^F]FDG followed previous dynamic [^18^F]FDG studies of brain metabolism (Lerche et al., 2008). Heads were stabilized on a mouldable pillow. Images were reconstructed with Ordered Subset Expectation Maximation with point spread function (3D-OSEM-PSF) modeling using 10 iterations.

### Tomography Procedures

We recorded all emission as 2D acquisitions. We completed the first tomography with [^11^C]PIB, 385 MBq (range 177–435 MBq), given intravenously as a 60-s 10 mL bolus dissolved in saline. We initiated the second tomography with injection of 207 MBq [^18^F]FDG (range 167–218 MBq) as a 30-s 5 mL bolus, also dissolved in saline.

### Magnetic Resonance Imaging

We acquired anatomical images for co-registration with the 3T Magnetom Tim Trio system (Siemens Healthcare, Erlangen, Germany) with 3D T1-weighted high-resolution anatomic scan of magnetization-prepared rapid acquisition gradient echo (MPRAGE) sequence.

### Motion Correction and Co-registration

We co-registered PET images with individual MR images to an MR template, and evaluated the quality of each co-registration by visual inspection in three planes. PET-to-MR correlated images were transformed into a common stereotaxic coordinate space (Talairach and Tournoux, 1988), and anatomical volumes of interest were used to extract time-activity-curves (TACs) from the dynamic PET images for the FDG and PIB analyses.

### Outcomes

The primary outcome was Aβ deposition as determined by [^11^C]PIB PET. Secondary outcome was the glucose metabolic rate measured with [^18^F]FDG. We also tested changes in cognitive capability.

### Kinetic Analysis of [^11^C]PIB Retention

We used the flow-independent Washout Allometric Reference Method (WARM) for analysis of washout and binding of [^11^C]PIB expressed as the tracer’s binding potential in brain (BP_ND_) (Rodell et al., 2013). We directly calculated the BP_ND_ without linearization with the operational equation,

$${BP}_{ND}(T)=\frac{m^{*}\overset{T}{\underset{0}{\int }}In[\frac{m_{ND}^{*}\left(t\right)}{m_{ND}^{*}\left(0\right)}]dt}{m_{ND}^{*}\overset{T}{\underset{0}{\int }}In[\frac{m^{*}\left(t\right)}{m^{*}\left(0\right)}]dt}-1$$

where m^∗^ and m_ND_^∗^ are measured PET signals as time-activity curves in regions with displaceable binding, and in a reference region, respectively. In this equation, the binding potential is corrected for flow differences, according to the initially deposited tracer, and the exponential nature of the washout.

### Cerebral Blood Flow

We used the rapid initial clearance of tracer PIB to obtain a surrogate absolute measure of cerebral blood flow (sCBF) (Rodell et al., 2013).

### Kinetic Analysis of [^18^F]FDG Uptake

Regional tissue time–activity curves for [^18^F]FDG uptake were extracted for eight predefined Regions-of-Interest (ROI). We calculated the net influx rate of [^18^F]FDG (K) from arterial blood samples and dynamic PET images applying multiple-times graphical analysis according to Gjedde (1982) and Patlak et al. (1983) for irreversible tracer uptake, with simple non-iterative perpendicular line fitting. Here, CMR_glc_ = K^∗^ C_a_/LC, where C_a_ is the arterial steady-state plasma glucose concentration, and LC is a common lumped constant value of 0.76, shown not to vary among the groups by calculation from kinetic parameters (Kuwabara et al., 1990).

### Regions-of-Interest (ROI)

In the analysis of [^11^C]PIB-PET, we included eight predefined areas of interest, the cingulate cortex, precuneus, frontal, parietal, temporal, and occipital lobes, and cerebellum and cerebral cortex. The cerebellar cortex was chosen as reference for the [^11^C]PIB retention measures obtained from parametric PET image maps by standard model based segmentation. For the analysis of the uptake of [^18^F]FDG, we included the same eight predefined areas in the analyses, to ensure that analyses covered the entire brain and that possible anatomical differences would be detected.

### Cognitive Testing

We evaluated cognition by the “Brief cognitive examination” from the Wechsler Memory Scale (WMS-IV) (Scheltens et al., 2010), the test examining orientation, time estimation, mental control, clock drawing, incidental recall, inhibition, and verbal reproduction.

### Statistical Analysis

The treatment period was set to 6 months, the period after which most clinical effects with liraglutide are achieved. The power calculation was based on a risk of type 1 error of 0.05 and risk of type 2 error of 0.20. We predicted differences of cerebral glucose consumption of 15% (Lerche et al., 2008) and changes of amyloid load of 15% (Leinonen et al., 2008) with SD set to 15%. The numbers yielded a sample size of *n* = 2 (1.96–0.84)^2^^∗^ 0.15^2^/0.15^2^ = 16. We analyzed treatment group data blindly by two-sample, Student’s two independent samples *t*-test to determine the significance of group differences, and by Student’s paired *t*-tests to determine changes within groups. *P*-values < 0.05 were considered indicative of significant difference.

## Results

We randomly assigned 38 patients with AD to receive either the GLP-1 analog liraglutide (*n* = 18) or placebo (*n* = 20). Fourteen patients in the liraglutide group and all patients in the placebo group completed the study. In the liraglutide group, 13 patients had PET with [^11^C]PIB, and 14 patients had PET with [^18^F]FDG, before and after treatment, compared to 19 with [^11^C]PIB, and 19 patients with [^18^F]FDG in the placebo group. All completed the cognitive examination. Tomography sessions were incomplete in two patients for CMR_glc_ and one patient for PIB binding, leaving 18 patients from the placebo group and 13 patients from the liraglutide group in the final analysis of [^11^C]PIB retention, and 17 patients from the placebo group and 14 patients from the liraglutide group in the final analysis of [^18^F]FDG uptake. Of the non-completers, one subject was excluded for drug-related reasons (nausea and anorexia after liraglutide for 35 days), and the rest for non-drug-related reasons that included; death of husband, one was diagnosed with cancer of the bladder which was evaluated by the principal investigator as being independent of the study drug treatment. One subject wanted to stop participation before randomization and one subject experienced nausea, vomiting, and fever after administration of 1 dose (0.6 mg) of the study drug which was evaluated by the principal investigator as being independent of the study drug treatment. All drop-outs had been treated with liraglutide.

### Demographic and Clinical Characteristics

Disease duration was longer in the liraglutide group (30 vs. 15 months, *P* = 0.015). Including dropouts in the analysis, the pattern remained significant (28 vs. 15 months, *P* = 0.018). There were no other significant differences at baseline (**Tables 2** and **3**). The liraglutide group was slightly younger (63.1 vs. 66.6 years, *P* = 0.16; or 65.4 vs. 66.6 years, *P* = 0.61 including drop-outs) and the female/male ratio was skewed. **Table 2** lists the demographic characteristics at completion.

**Table 2:** Demographic and baseline characteristics for completers at randomisation.

	Placebo group	Liraglutide group	*P*
	mean (range; SEM)	mean (range; SEM)	
*n*	20	14	
Age, y	66.6 (50–80; 1.8)	63.1 (55–70; 1.3)	0.16
Sex	15Male/5Female	6 Male/8 Female	
Gender (% female)	25	57	-
Wechsler Memory Scale	27.2 (0–57; 3.8)	27.1 (5–44; 3.4)	0.99
Duration of Alzheimer’s disease (AD), months	15 (1–41; 2.5)	29.5 (5–70; 5.8)	0.015^∗^
Onset, years	65.3 (49–77; 1.9)	60.3 (50–67; 1.4)	0.054


*Data are presented as mean range and standard error of the mean (SEM), and p-values were calculated using unpaired t-test. The Wechsler Memory Scale (WMS-IV) score provides a single score of overall cognitive performance; ^∗^*P* < 0.05.*

**Table 3:** Patient characteristics.

Baseline	Placebo group (*n* = 20)	Liraglutide group (*n* = 14)	Placebo vs. liraglutide group	
	mean (SEM)	mean (SEM)	*P*	

Fasting plasma-glucose (mmol/L)	5.8 (0.2)	5.6 (0.2)	0.24	
Glycated hemoglobin (HbAlc)	0.056 (0.001)	0.055 (0.0006)	0.41	
Plasma-cholesterol total(mmol/L)	5.9 (0.3)	5.7 (0.2)	0.67	
Weight (kg)	77.8 (3.0)	74.1 (2.1)	0.38	
Body Mass Index (BMI) (kg/m	25.1 (0.7)	25.1 (1.0)	0.96	
Systolic blood pressure (mmHg)	149.7 (4.3)	144.1 (6.3)	0.46	
Diastolic blood pressure (mmHg)	87.5 (2.4)	83.9 (2.5)	0.32	
Heart rate (bpm)	62.7 (3.2)	64.8 (2.8)	0.64	

**After 6 Months**	**Placebo group (*n* = 20)**	**Liraglutide group (*n* = 14)**	**Placebo vs. liraglutide group**	**Change placebo vs. change liraglutide group**
	**mean (SEM)**	**mean (SEM)**	***P***	***P***

Fasting plasma-glucose (mmol/L)	5.6 (0.1)	5.1 (0.1)	0.0041*	0.10
Glycated hemoglobin (HbAlc)	0.056 (0.0008)	0.054 (0.0008)	0.23	0.68
Plasma-cholesterol total (mmol/L)	5.5 (0.3)	5.1 (0.2)	0.27	0.12
Weight (kg)	76.1 (2.8)	69.2 (2.0)	0.071	0.0083*
Body Mass Index (BMI) (kg/m	24.6 (0.7)	23.4 (0.9)	0.26	0.0069*
Systolic blood pressure (mmHg)	151.4 (4.8)	132.4 (5.6)	0.015*	0.013*
Diastolic blood pressure (mmHg)	88.8 (2.7)	80.3 (2.5)	0.035*	0.19
Heart rate (bpm)	68.4 (4.3)	66.1 (1.8)	0.67	0.46


*Data are presented as mean (SEM), and P-value is calculated using paired/independent samples t-test. ^∗^*P* < 0.05.*

We found a significant difference in fasting plasma glucose between the two groups (5.6 mM in the placebo group and 5.1 mM in the GLP-1 analog group, *P* = 0.0041) after 6 months; HbA1c remained unchanged. Weight decreased in the liraglutide group during the first 3 months, hereafter weight curves were parallel (weight loss at 6 months: 4.9 kg vs. 1.6 kg, *P* < 0.01). We observed a significant reduction in systolic and diastolic blood pressure in the liraglutide group at the end of the study period (*P* = 0.015 and *P* = 0.035, respectively.) We found no significant difference in heart rate between the two groups at the end of the study (*P* = 0.67). Patient characteristics at baseline and 6 months are listed in **Table 3**.

### Parametric PET Measures

#### [^11^C]PIB Retention (**Figure 1**)

**FIGURE 1:**
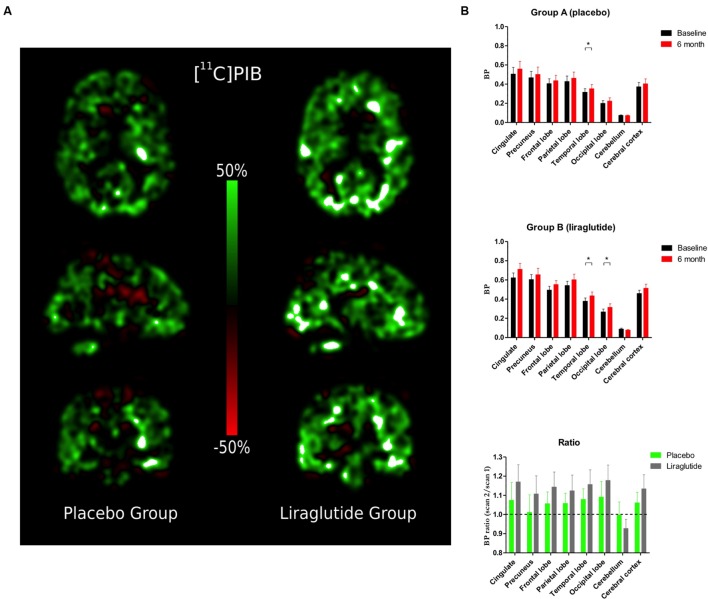
**(A)** Percentage change in Binding Potential in the brain (BP_ND_) between baseline scan and 6-month follow-up. **(B)** Binding Potential in the brain (BP_ND_) in the cingulate cortex, precuneus, frontal, parietal, temporal, and occipital lobes, cerebellum and cerebral cortex at baseline and the Ratio Session 2/1 in the placebo group and the liraglutide group.^∗^*P* < 0.05.

Percentage changes of ligand binding potential in brain (BP_ND_) between the baseline and the 6-month follow-up are shown in **Figure 1A**. [^11^C]PIB binding (**Figure 1B**) increased significantly by the folllowing mean ratio(s) in temporal lobe (*P* = 0.04, 0.036, 95% CI: 0.0012; 0.070), and numerically but insignificantly in occipital lobe (*P* = 0.09, 0.023, 95% CI: -0.0043; 0.050), in the placebo group brains. In liraglutide group brains, [^11^C]PIB binding increased significantly by the following mean ratios in the temporal (*P* = 0.04, 0.056, 95% CI: 0.0017; 0.11) and occipital (*P* = 0.04, 0.048, 95% CI: 0.0013; 0.094) lobes. Also in the GLP-1 analog treatment group, we found numerical but insignificant increases by the following mean ratios in the cingulate (*P* = 0.09, 0.088, 95% CI: -0.017; 0.19) and cerebral (*P* = 0.09, 0.055, 95% CI: -0.0099; 0.12) cortices, and a numerical but insignificant mean ratio decrease in cerebellum (*P* = 0.09, 0.010, 95% CI: 0.023; -0.0019). The ratios of the mean post-treatment [^11^C]PIB retentions to the pre-treatment baseline in eight ROIs are shown in **Figure 1B**. The average session 2/session 1 ratios were not significantly different between the groups (*P* ≥ 0.38) in any region.

Using the rapid initial clearance of [^11^C]PIB as a surrogate absolute measure of sCBF, we found that sCBF increased in the placebo group but not in the liraglutide group. In the placebo group, sCBF increased significantly by the following means in the frontal (*P* = 0.04, 3.56 ml/hg/min, 95% CI: 0.17; 6.94), parietal (*P* = 0.04, 3.27 ml/hg/min, 95% CI: 0.10; 6.45), and occipital (*P* = 0.02, 3.27 ml/hg/min, 95% CI: 0.10; 6.45) lobes, and in cerebellum (*P* = 0.009, 4.47 ml/hg/min, 95% CI: 1.28; 7.65), and in cortex as a whole (*P* = 0.03, 3.61 ml/hg/min, 95% CI: 0.41; 6.81). Numerical but insignificant mean increases were noted in cingulate cortex (*P* = 0.08, 3.40 ml/hg/min, 95% CI: -0.48; 7.28), and in precuneus (*P* = 0.055, 3.99 ml/hg/min, 95% CI: -0.10; 6.45). No significant changes occurred in liraglutide group brains (*P* ≥ 0.41), or of the ratios between the two groups (*P* ≥ 0.13).

#### Cerebral Glucose Metabolism (**Figure 2**)

**FIGURE 2:**
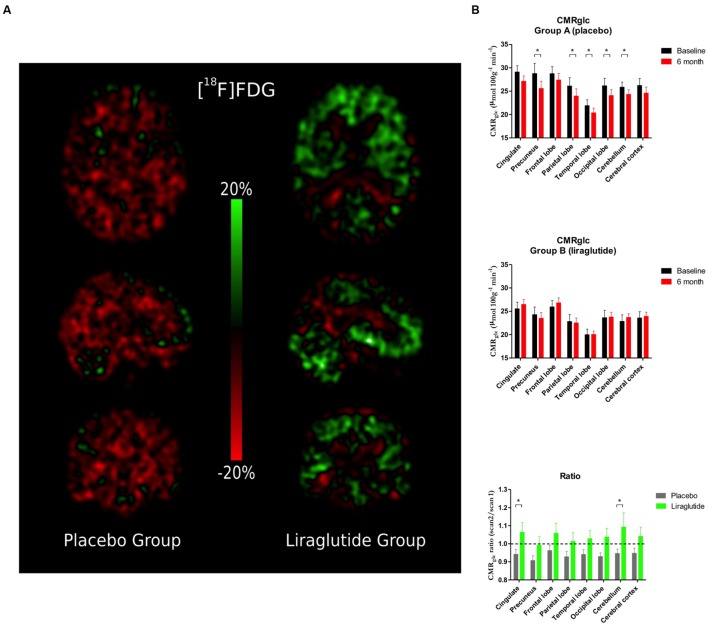
**(A)** Percentage change in cerebral metabolic rate for glucose (CMR_glc_) between baseline scan and 6-month follow-up. **(B)** Cerebral metabolic rate for glucose uptake (CMR_glc_) in the cingulate cortex, precuneus, frontal, parietal, temporal, and occipital lobes, cerebellum, and cerebral cortex at baseline and the Ratio Session 2/1 in the placebo group and the liraglutide group. ^∗^*P* < 0.05.

Percentage changes of CMRglc between baseline and 6-month follow-up are shown in **Figure 2A**. In the placebo group, we observed significant decreases of CMR_glc_ by the following means in precuneus (*P* = 0.009, 3.2 μmol/hg/min, 95% CI: 5.45; 0.92), and in parietal (*P* = 0.04, 2.1 μmol/hg/min, 95% CI: 4.21; 0.081), temporal (*P* = 0.046, 1.54 μmol/hg/min, 95% CI: 3.05; 0.030), and occipital (*P* = 0.009, 2.10 μmol/hg/min, 95% CI: 3.61; 0.59) lobes, and in cerebellum (*P* = 0.04, 1.54 μmol/hg/min, 95% CI: 3.01; 0.064). We noted non-significant mean decreases in the cingulate cortex (*P* = 0.05, 1.98 μmol/hg/min, 95% CI: 4.00; -0.042) and in cerebral cortex as a whole (*P* = 0.08, -1.64 μmol/hg/min, 95% CI: 3.53; -0.25). In the GLP-1 analog group, the CMR_glc_ increased numerically but non-significantly (all *P* ≥ 0.49) as shown in **Figure 2B**. In the placebo group, the session 2/session 1 ratios of CMR_glc_ in the cingulate (*P* = 0.04) and occipital lobes (*P* = 0.04) declined significantly compared to the liraglutide group.

### Cognitive Outcome

We found no significant differences from baseline in total cognitive scores after treatment within, or between, the two groups (**Table 4**). Average scores at baseline were 27.1 in the liraglutide group, and 27.2 in the placebo group (*p* = 0.99) and we found no significant differences from baseline in total cognitive score after treatment within, or between the two groups (liraglutide 0.43, placebo 1.7; *P* = 0.50). A significant impairment of the orientation test result was found in the placebo group (*P* = 0.041). The scores of the liraglutide group participants did not change.

**Table 4:** Cognitive outcome.

Wechsler Memory Scale (WMS-IV)	Placebo group (*n* = 20)		Liraglutide group (*n* = 14)		Change placebo vs. change liraglutide group
			
	Baseline	After 6 months	Baseline	After 6 months	
	mean (*SD*)	mean (*SD*)	mean (*SD*)	mean (*SD*)	*P*
Orientation)	3.0 (3.2)	1.3 (2.2)^∗^	1.9 (3.1)	2.0 (2.9)	0.085
Time estimation	1.7 (1.6)	2.0 (1.8)	1.7 (1.5)	1.2 (1.2)	0.20
Mental control	4.8 (4.9)	4.7 (4.9)	4.9 (4.6)	4.9 (4.7)	0.93
Clock drawing	2.7 (1.4)	2.7 (1.5)	2.2 (1.6)	2.6 (1.3) ^†^	0.25
Incidental recall	2.4 (3.1)^¥^	1.9 (3.0)	0.57 (1.2)	0.14 (0.53)^‡^	0.88
Inhibition	9.9 (5.7)	10.6 (6.1)	12.4 (4.8)	11.9 (5.0)	0.10
Verbal reproduction	2.7 (2.5)	2.4 (2.5)	3.4 (2.5)	3.9 (2.4)	0.34
**Total score**	**27.2 (17.0)**	**25.5 (17.0)**	**27.1 (12.8)**	**26.6 (13.0)**	**0.50**


*Tests for orientation, time estimation, mental control, clock drawing, incidental recall, inhibition, and verbal production are done using Mann–Whitney test. Total score are done using independent samples t-test. All data presented as means ± SD. ^∗^Visit2 > Visit5, *P* = 0.041; ^†^Visit5 > Visit2, *P* = 0.15; ^¥^Placebo group > liraglutide group for Visit 2, *P* = 0.064; ^‡^Visit5 < Visit2, *P* = 0.083.*

## Discussion

At present, AD is treated symptomatically, and current therapeutics target neurotransmission rather than neurodegeneration or neuronal metabolism. This study tested the hypothesis that treatment with the GLP-1 analog liraglutide, a drug that potentially affects neurodegeneration, neuronal performance, and neuroinflammation, would reduce intracerebral Aβ deposition and improve glucose metabolism in the CNS of patients with AD, followed by improvement of cognition. The analog prevented the decline of brain glucose consumption, but we found no effect on fibrillary Aβ accumulation or cognition. The size of the cohort and the duration of the study, however, precluded definite clinical conclusions but the results encouraged further investigations.

### [^11^C]PIB Retention

A conventional explanation of the pathogenesis of AD is the Aβ cascade, according to which increased production or decreased clearance of fibrillary Aβ leads to loss of dendritic spines and symptoms of dementia (Hardy and Higgins, 1992). Patients with fibrillary Aβ deposits have statistically greater retention of [^11^C]PIB than patients without the Aβ deposits, and the binding correlates with amyloid deposits in brain tissue removed at autopsy (Leinonen et al., 2008). Here, we observed a slight but insignificant numerical increase of the estimates of amyloid accumulation in all cortical areas after the 6 months of treatment, with no differences between the two groups.

In contrast, animal studies have shown significant changes in Aβ load after treatment with the GLP-1 analog liraglutide (McClean et al., 2011). The present patients therefore may be at the stage of the disease at which [^11^C]PIB binding no longer undergoes the increases seen in the animal models (Engler et al., 2006; Kadir et al., 2012). A direct correlation between disease progression and [^11^C]PIB retention has not been demonstrated. As suggested by the US NIA-Alzheimer’s Association, biomarkers of AD (hyperphosphorylated tau and Aβ) have the necessary specificity for a diagnosis of AD, whereas tracers of brain metabolic changes such as [^18^F]FDG serve to assess disease progression and treatment effect (Dubois et al., 2014). Thus, the finding was negative for the primary outcome, but we cannot exclude an effect of liraglutide on Aβ accumulation to a significant degree, such as predicted in the power calculations for this study of patients at an earlier stage. The number analyzed (*n* = 14) approached but did not reach the number obtained by power calculation (*n* = 16), leading us to be cautious with conclusions of this variable.

In three studies, Hölscher and colleagues reported that GLP-1 mimetics have prophylactic effects and reduce Alzheimer-like disease progression in the APP/PS1 mouse model. The reduction was most pronounced in the early stages of this animal model of AD (McClean et al., 2015), compared to middle-aged mice with disease advanced to a stage where the first behavioral symptoms appear (McClean et al., 2011), and in aged APP/PS1 mice representing the late stage of AD (McClean and Holscher, 2014). The randomization in the present study resulted in significantly longer duration of AD in the liraglutide treated group of patients. The different disease durations and the short period of treatment may have blunted a significant effect of the GLP-1 analog on the disease marker at the earlier stage.

The sCBF measures gleaned from the [^11^C]PIB retention increased in the placebo group, but the implications of the increase are not clear. Recently, Lacalle-Aurioles et al. (2014) noted an inverse relation between CBF and cortical thickness that suggested an increase of CBF associated with cortical atrophy in pre-dementia stages of AD. Such increases would tend to rule out deficient oxygen or glucose delivery as explanations of the declining metabolism of glucose (Rodell et al., 2012).

### Cerebral Glucose Metabolism (CMR_glc_)

As a pre-specified secondary outcome, we measured CMR_glc_. Reductions of CMR_glc_ commonly are correlated with cognitive decline in patients with AD, in particular in the parietotemporal, frontal, and posterior cingulate cortices (Mosconi, 2005; Engler et al., 2006; Nordberg et al., 2010), and PET with [^18^F]FDG reveals the progressive reduction in cerebral glucose metabolism in patients with subsequent pathologically proven AD before clinical symptoms are detected (Nordberg et al., 2010).

In the precuneus and parietal, temporal, and occipital cortices, and in cerebellum, participants treated with placebo had significantly decreased CMR_glc_ after 6 months, in agreement with PET determinations of glucose metabolism in AD that show a consistent pattern of reduced cerebral glucose utilization beginning in parietal and temporal regions, later spreading to prefrontal cortices (Small et al., 2000). Also, the magnitude of CMR_glc_ decrease is compatible with previous reports (Alexander et al., 2002). The [^18^F]FDG metabolite retention in brain is sensitive to disease progression, and decline is closely related to cognitive impairment (Dubois et al., 2014). In contrast, patients treated with liraglutide had numerical but statistically insignificant increases of CMR_glc_ after 6 months, implying that the GLP-1 analog treatment prevented the decline of CMR_glc_ that reflects cognitive impairment, synaptic dysfunction, and disease progression, despite longer disease duration in this group.

Little is known of the effects of GLP-1 and analogs on brain metabolism, but the present results are in line with previously documented effects of GLP-1 on cerebral glucose metabolism (Gejl et al., 2012, 2013). Multiple physiological mechanisms may explain this observation: The brain import of glucose across the blood-brain barrier occurs by means of stereospecific and non-energy-demanding facilitated diffusion, mediated by the glucose transporter GLUT1 (Gjedde, 1983). The number of glucose transporters is reduced in AD (Simpson et al., 1994; Mooradian et al., 1997), and accelerated amyloid load and aggravated Aβ accumulation recently were shown in a transgenic mouse model of AD with GLUT1 deficiency (Winkler et al., 2015). Native GLP-1 may have a direct activating effect on transport by GLUT1 in brain capillary endothelium (Gejl et al., 2013, 2014), by which mechanism liraglutide may be expected to prevent the decline in glucose uptake in AD.

It is known that a decline of blood–brain barrier transport of glucose fails directly to limit brain glucose metabolism that is regulated by hexokinase activity (Gejl et al., 2014). Recently, restoration of cerebral and systemic microvascular architecture by liraglutide treatment was reported to prevent further microvascular degeneration in AD and decline of glucose transport (Kelly et al., 2015). Molecular links exist between reduced insulin signaling in brain of AD patients, and peripheral insulin signaling in patients with diabetes have both been established (Bomfim et al., 2012), and liraglutide has been reported to prevent this insulin desensitization in the brain and to reverse insulin signaling impairments in human AD brain tissue (Talbot et al., 2011). As demonstrated by Craft et al. (2012), insulin administered intranasally raises CMR_glc_ in AD. Therefore, the activation by liraglutide of insulin-related pathways is a possible explanation of the results (Freiherr et al., 2013).

Diminished glucose metabolism characterizes AD and correlates with impaired cognition (Hunt et al., 2007). The advanced stage of AD in this study along with the uneven randomization regarding disease duration may have led to an underestimation of the effects of treatment. Future clinical trials focusing on patients with mild cognitive impairment may prove more effective in specifically delineating the glucose-related effects of liraglutide on cognition.

### Cognition

Animal studies suggest that cognition may be improved by liraglutide (Hansen et al., 2015). In the present study, cognitive testing revealed no significant decrease of WMS score in either group after 6 months of treatment with liraglutide versus placebo, although negative changes of cognition over time seemed smaller in the liraglutide-treated group. A significant impairment in the orientation test was found in the placebo group and not in the liraglutide group, but the study was underpowered for this outcome. Thus, the cognitive assessment was not definitive.

### Side Effects

The risk of hypoglycaemia is very low for liraglutide as its effects on insulin secretion are glucose dependent. No subject experienced side effects related to hypoglycaemia and HbA1c levels were unaffected.

The most common side effects reported in patients treated with liraglutide are gastrointestinal, mostly transient nausea, as also observed here. As expected, most cases of nausea occurred during the first 4 weeks. One dropout in the liraglutide group was due to side effects.

Moderate weight loss has been reported in diabetes patients treated with liraglutide. A significant weight loss was seen in the liraglutide group. The weight loss abated after 2–3 months of treatment. The weight reduction with GLP-1 agonists is predominantly due to a reduction in adipose tissue, especially visceral adipose tissue, rather than to a reduction in muscle mass (Jendle et al., 2009). Weight loss, the main side effect, must be evaluated closely in future studies, however. Whether liraglutide-induced changes in weight or BMI can be linked to cerebral glucose metabolism or other outcome variable of the patients remains to be determined, as a clear link between cerebral and peripheral insulin resistance has yet to be established.

We found a significant decrease in systolic blood pressure after 6 months of GLP-1 analog treatment, compared to the placebo group. We found no change in heart rate at the end of the study period in either group.

## Conclusion

In AD patients with long-standing disease, 26 weeks of liraglutide treatment prevented the expected decline of CMR_glc_ that reflects disease progression, as observed in the placebo group. We found no differences between the groups treated with liraglutide and placebo with respect to amyloid deposition or cognition.

## Author Contributions

LE, JR, and BB conceived the study. BB, AG, AM, BB, and JR designed the study. MG, AG, SH, KV, AR, and AS did imaging data preprocessing and statistical analysis. NM contributed reagents/materials/analysis tools. LE, HB, HG, BB, and JR did patient assessments. LE, MG, AG, KV, and AR did the statistical analysis for the report. MG, AG, BB, and JR wrote the report. All authors contributed to the subsequent drafts and approved the final version. LE and MG contributed equally to the study.

## Conflict of Interest Statement

MG: None. AG: Advisory panel; Executive Council, European Dana Alliance for the Brain, European Research Council. Consultant; National Expert, Horizon2020 SC1 PC Health, European Commission, Novo-Nordisk A/S. Research support; Danish Council of Independent Research, National Institutes of Health BRAIN Initiative. Speakers bureau; MINDexult. Stocks/shareholder; Danske Bank. LE: None. AM: None. SB Hansen: None. KV: None. AR: None. HB: None. HG: None. AS: None. NM: None. BB: Board Member; Author; Allergan, Inc., Novo Nordisk A/S. Research Support; Author; Novo Nordisk A/S. Speaker’s Bureau; Author; Pfizer Inc. JR: Advisory Panel; Author; Merck & Co., Inc., Novo Nordisk A/S, Eli Lilly and Company, Sanofi U.S. Employee; Spouse/Partner; Novo Nordisk A/S. Research Support; Author; Novo Nordisk A/S, Eli Lilly and Company, Amylin Pharmaceuticals, LLC. Speaker’s Bureau; Author; Merck & Co., Inc., Novo Nordisk A/S, Johnson & Johnson, Sanofi U.S., AstraZeneca/Bristol-Myers Squibb.
